# Complete mitochondrial genome of *Schizothorax gongshanensis* (Cypriniformes: Cyprinidae)

**DOI:** 10.1080/23802359.2016.1172272

**Published:** 2016-07-23

**Authors:** Weiwen Li, Yang Liu, Qianghua Xu

**Affiliations:** a College of Marine Sciences, Shanghai Ocean University, Shanghai, P.R. China;; b Key Laboratory of Sustainable Exploitation of Oceanic Fisheries Resources, Ministry of Education, Shanghai, P.R. China;; c National Engineering Research Center for Oceanic Fisheries, Shanghai, P.R. China;; d Collaborative Innovation Center for Distant-Water Fisheries, Shanghai Ocean University, Shanghai, P.R. China

**Keywords:** Mitochondrial genome, *Schizothorax gongshanensis*, stock assessment

## Abstract

In this study, the complete mitochondrial DNA of *Schizothorax gongshanensis* was determined. The complete genome is 16,591 bp in length which contains 13 protein-coding genes, 22 transfer RNA genes, 2 rRNA genes, an origin of light-strand replication (O_L_) and a control region (D-Loop). The complete mtDNA sequence of *S. gongshanensis* provides a useful genetic marker for the studies on molecular systematics, population genetics and stock assessment.

*Schizothorax gongshanensis* is an important fishery species and mainly distributed in Gongshan area, located in the upstream of Nujiang River (Chen 2013). Due to overfishing, environment pollution and habitat degradation, the population of this species has collapsed (reported by local fishermen). The basic biological information of this species is of significance in developing effective strategies for management of this resource. However, only a few reports about the information on the species' population, biology and ecology. For example, shrub’s blades and other foreign C3 plants were reported to make a great contribution to the *S. gongshanensis* feeding ecology (Zhou 2009). The complete mitochondrial DNA of *S. gongshanensis* is still unknown. It is undoubted that the complete mitochondrial genome data of the *S. gongshanensis* will be useful for studying on the population history, phylogeography and stock assessment, which will ultimately improve the fishery stock management of the *S. gongshanensis.*

In this study, *S. gongshanensis* individuals were collected from Gongshan district (27°03′N 98°52′E), the upstream of Nujiang River. The muscles (near to dorsal fin) of the sample were kept in 95% alcohol for shipping to the laboratory (Key Laboratory of Sustainable Exploitation of Oceanic Fisheries Resources, Ministry of Education). The complete mitogenome sequence of *S. gongshanensis* was determined. Primers for PCR amplification and sequencing were designed according to the mtDNA sequence of *Schizothorax biddulphi* (GenBank Accession No. NC_017873) (Gong et al. 2012), a close species of *S. gongshanensis*. After assembling and alignment, the complete mitogenome sequence of *S. gongshanensis* was deposited into GenBank database with accession number (KT946652). The whole length of mitogenome is 16,591 bp, which contains 13 protein-coding genes, 22 tRNA genes, 2 rRNA genes, a rep-region and a control region (details shown in GenBank KT946652). The organization structural of mitogenome is similar with other *Schizothorax* (Chen et al. 2013; Goel et al. 2014).

A neighbor-joining (NJ) tree (Figure 1) was constructed to represent the phylogenetic relationships between *S. gongshanensis* and other *Schizothorax* species. The complete mitochondrial DNA sequences of *Schizothorax yunnanensis paoshanensis* (KP892531), *Schizothorax nukiangensis* (KT223584), *Schizothorax lantsangensis* (KP143725), *Schizothorax dolichonema* (KJ577589), *Schizothorax kozlovi* (KJ755668), *Schizothorax prenanti* (KJ126773), *Schizothorax chongi* (KJ718889), *Schizothorax lissolabiatus* (KP796150), *Schizothorax davidi* (KM879227), *Schizothorax macropogon* (KC020113), *Schizothorax waltoni* (KC513574), *Schizothorax oconnori* (KC513575), *Schizothorax wangchiachii* (KC292197), *Schizothorax biddulphi* (JQ844133), *Schizothorax progastus* (KF739399), *Schizothorax richardsonii* (KC790369), *Schizothorax labiatus* (KF739398) and *Schizothorax esocinus* (KT210882) were obtained from GenBank, which were closely related to the *S. gongshanensis* were used to construct the NJ tree using the Kimura 2-parameter model by MEGA 4.0 (Tamura et al. 2007). As shown in Figure 1, *S. yunnanensis paoshanensis* and *S. nukiangensis* are the most closely related species of the *S. gongshanensis* among all the 19 species used for Neighbor-Joining tree construction, with full statistical reliability (bootstrap value, 100%). While *S. richardsonii* and *S. esocinus* exhibited the farthest genetic relationships with *S. gongshanensis* according to the sequences included in this study.

**Figure 1.**
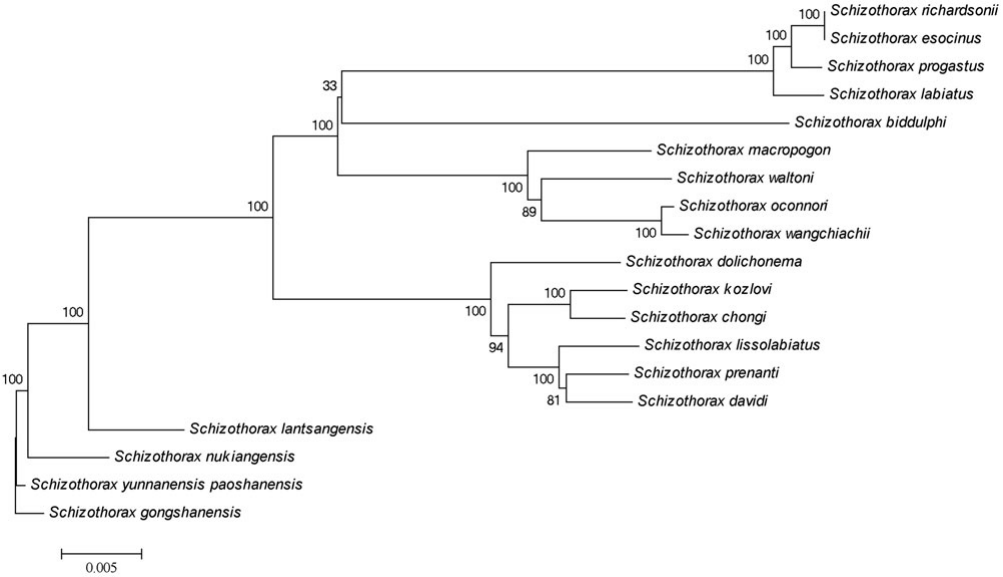
The neighbor-joining tree of 19 schizothoracinae species. A neighbor-joining tree was constructed to represent the phylogenetic relationships between *S. gongshanensis* and other Schizothorax species. The complete mitochondrial DNA sequences of *Schizothorax yunnanensis paoshanensis* (KP892531), *Schizothorax nukiangensis* (KT223584), *Schizothorax lantsangensis* (KP143725), *Schizothorax dolichonema* (KJ577589), *Schizothorax kozlovi* (KJ755668), *Schizothorax prenanti* (KJ126773), *Schizothorax chongi* (KJ718889), *Schizothorax lissolabiatus* (KP796150), *Schizothorax davidi* (KM879227), *Schizothorax macropogon* (KC020113), *Schizothorax waltoni* (KC513574), *Schizothorax oconnori* (KC513575), *Schizothorax wangchiachii* (KC292197), *Schizothorax biddulphi* (JQ844133), *Schizothorax progastus* (KF739399), *Schizothorax richardsonii* (KC790369), *Schizothorax labiatus* (KF739398) and *Schizothorax esocinus* (KT210882) were used to construct the NJ tree using the Kimura 2-parameter model by MEGA 4.0.
